# Transcriptional Control in Cardiac Progenitors: Tbx1 Interacts with the BAF Chromatin Remodeling Complex and Regulates *Wnt5a*

**DOI:** 10.1371/journal.pgen.1002571

**Published:** 2012-03-15

**Authors:** Li Chen, Filomena Gabriella Fulcoli, Rosa Ferrentino, Stefania Martucciello, Elizabeth A. Illingworth, Antonio Baldini

**Affiliations:** 1 Texas Heart Institute, Houston, Texas, United States of America; 2 Institute of Genetics and Biophysics “Adriano Buzzati Traverso,” National Research Council, Naples, Italy; 3 Department of Chemistry and Biology, University of Salerno, Fisciano, Italy; 4 University Federico II, Naples, Italy; Stanford University School of Medicine, United States of America

## Abstract

Mutations of the *Wnt5a* gene, encoding a ligand of the non-canonical Wnt pathway, and the *Ror2* gene, encoding its receptor, have been found in patients with cardiac outflow tract defects. We found that *Wnt5a* is expressed in the second heart field (SHF), a population of cardiac progenitor cells destined to populate the cardiac outflow tract and the right ventricle. Because of cardiac phenotype similarities between *Wnt5a* and *Tbx1* mutant mice, we tested potential interactions between the two genes. We found a strong genetic interaction in vivo and determined that the loss of both genes caused severe hypoplasia of SHF–dependent segments of the heart. We demonstrated that *Wnt5a* is a transcriptional target of Tbx1 and explored the mechanisms of gene regulation. Tbx1 occupies T-box binding elements within the *Wnt5a* gene and interacts with the Baf60a/Smarcd1 subunit of a chromatin remodeling complex. It also interacts with the Setd7 histone H3K4 monomethyltransferase. Tbx1 enhances Baf60a occupation at the *Wnt5a* gene and enhances its H3K4 monomethylation status. Finally, we show that Baf60a is required for Tbx1–driven regulation of target genes. These data suggest a model in which Tbx1 interacts with, and probably recruits a specific subunit of, the BAF complex as well as histone methylases to activate or enhance transcription. We speculate that this may be a general mechanism of T-box function and that Baf60a is a key component of the transcriptional control in cardiac progenitors.

## Author Summary

We have demonstrated a novel interaction between the *Tbx1* gene, the mutation of which causes DiGeorge syndrome, and *Wnt5a*, another human disease gene, which is important for oriented cell migration and cell polarity. We found that, in mice, reduced dosage of each of the two genes enhances the phenotype caused by the mutation of the other. Loss of the two genes in mice has very severe consequences for heart development. Our genetic and biochemical data determined that Tbx1, a transcription factor of the T-box family, regulates *Wnt5a* expression. We found that Tbx1 targets the BAF chromatin remodeling complex to the *Wnt5a* gene and interacts with a histone monomethyltransferase. *Tbx1* expression increases Baf60a occupation of the *Wnt5a* gene and enhances its H3K4 monomethylation status, while Baf60a knockdown abolishes the ability of Tbx1 to regulate *Wnt5a* and other target genes. Overall, our data identify *Wnt5a* as an important effector of Tbx1 function in heart development and demonstrate that Tbx1 regulates the gene by interacting with the chromatin remodeling and histone methylation machinery.

## Introduction

The second heart field (SHF) provides progenitor cells for the development of several segments of the mature heart, such as the outflow tract, right ventricles and atria [1], [2]. Mouse models of congenital heart disease suggest that perturbation of SHF development may be the basis of relatively common heart defects in humans, such as conotruncal anomalies, but the transcriptional mechanisms driving SHF development are not well defined. An important example of a congenital heart disease gene that functions within the SHF is *TBX1* encoding a T-box transcription factor. This is haploinsufficient in the DiGeorge/Velocardiofacial/22q11.2 deletion syndrome, which is associated with various types of cardiac outflow tract (OFT) and vascular defects [3]. *Tbx1* mouse mutants recapitulate well the human phenotype, which has facilitated a detailed analysis of the role of the gene in heart development. In particular, *Tbx1* is expressed in the SHF where it sustains cell proliferation and inhibits differentiation [4]. However, there is considerable less information about the effectors of these developmental roles, and about the mechanisms for target gene regulation.

SHF cardiac progenitors, which reside outside the heart, are thought to migrate into the heart and differentiate as they are incorporated into the OFT. Thus, it is reasonable to expect that transcriptional regulation of SHF progenitors should involve cell polarity and cell migration, functions that in many cases are regulated by the non-canonical Wnt pathway [5]. Here we show that *Wnt5a*, which encodes a ligand of the non-canonical pathway, is expressed in the SHF. It has been shown that *Wnt5a* ^−/−^ animals have OFT abnormalities, though generally not as severe as in *Tbx1* ^−/−^ animals [6]. In addition, OFT defects have also been shown in *Ror2* ^−/−^ mice, Ror2 being a receptor of Wnt5a. Furthermore, mutations of *WNT5a* or *ROR2* in humans are associated with Robinow syndrome [7], which includes OFT defects, though at a low penetrance. Therefore, we postulated that there may be interaction between Tbx1 and the non-canonical Wnt pathway. We have crossed *Tbx1* and *Wnt5a* mouse mutants and found that there is indeed a genetic interaction and, most interestingly, the loss of both genes caused developmental failure of the SHF-dependent heart segments, indicating that the two genes are required for SHF function. Next, we investigated the transcriptional mechanisms underlying this interaction. We found that *Wnt5a* is a transcriptional target of Tbx1. Previous data showing a genetic interaction between *Tbx1* and the gene encoding the chromodomain protein Chd7 [8] and physical interaction with the histone methyltransferase Ash2l [9] prompted us to investigate the role of chromatin remodeling and histone modifiers. We found that Tbx1 co-immunoprecipitates with Baf60a, a component of the SWI-SNF-like BAF chromatin remodeling complex, and with the Setd7 histone 3, Lysin 4 monomethyltransferase. Tbx1 expression increases the occupation of the *Wnt5a* gene by Baf60a, and enhances the H3K4 monomethylation status of the chromatin in the T-box binding element (TBE)-harboring regions of the *Wnt5a* gene. Finally, we found that Baf60a is required for Tbx1-induced regulation of *Wnt5a*. Overall, our data support a mechanism by which Tbx1 enhances transcription of *Wnt5a* (and possibly other target genes) by interacting with a chromatin remodeling complex and enhancing H3K4 de novo methylation.

This is the first evidence of an impact of Tbx1 on chromatin status and of its interaction with the BAF complex. Our data also raises the intriguing possibility that Baf60a is a critical BAF subunit for the transcriptional program of cardiac progenitors.

## Results

### *Wnt5a* is regulated by Tbx1 in the SHF

**Figure 1:**
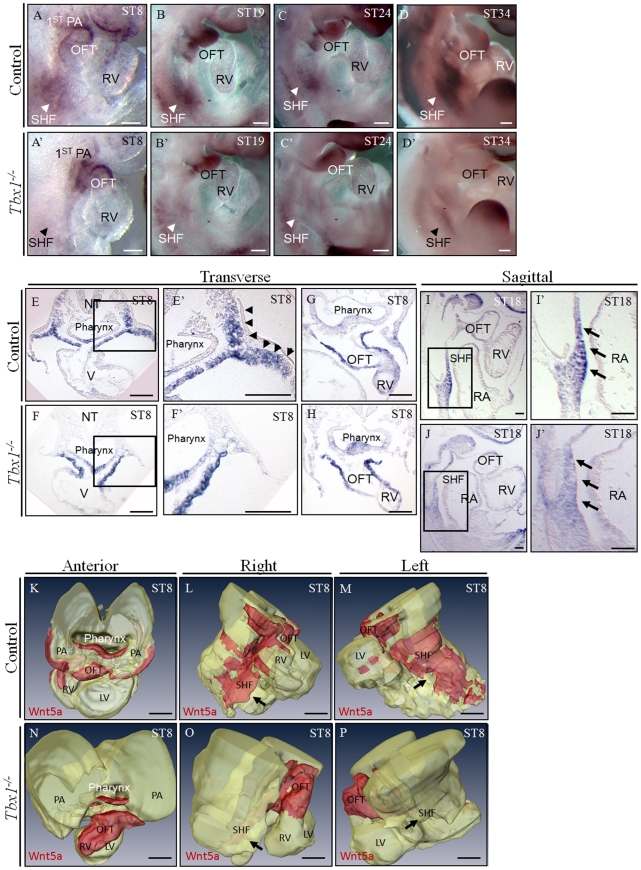
*Wnt5a* is expressed in the second heart field (SHF) and is downregulated in *Tbx1^−/−^* embryos. (A–D′) The expression of *Wnt5a* was downregulated in the SHF, but not in the outflow tract (OFT) or the pharyngeal arch (PA) core mesoderm of *Tbx1^−/−^* embryos at E8.0–11.5 (A′–D′), compared with the littermate controls (A–D), shown by whole-mount *in situ* hybridization (n = 3). Right view; arrowheads indicate the SHF. (E–J′) Transverse or sagittal sections cut from whole-mount *in situ* hybridized E8.0–9.0 *Tbx1^−/−^* embryos (F, H, J) showed the downregulation of *Wnt5a* expression, compared with the littermate control (E, G, I, n = 3); squared regions are magnified on the adjacent panels; arrows indicate the SHF/splanchnic mesoderm domain. Arrowheads on panel E′ indicate a mesodermal expression domain strongly affected by the loss of *Tbx1*. (K–P) 3D reconstructions of E8.0 WT and *Tbx1^−/−^* embryos provide a schematic, overall view of the expression domains regulated by *Tbx1*. Reconstructions were built using sections from embryos shown in E and F. Arrows indicate the SHF. PA: pharyngeal arch; OFT: outflow tract; SHF: second heart field; RV: right ventricle; ST: somite; NT, notochord; RA, right atrium; V: common ventricle; LV, left ventricle. Scale bars: 100 µm.

We carried out in situ hybridization of *Wnt5a* in embryos from E8.0 to E11.5 and found strong expression in the pharyngeal and splanchnic mesoderm including the SHF region (Figure 1). In addition, we found expression in the OFT, as previously noted [6], [10]. We next tested whether *Wnt5a* expression may be altered by *Tbx1* mutation. At E8.0–8.5, *Wnt5a* expression was reduced in the SHF of *Tbx1* ^−/−^ embryos (n = 3; Figure 1), while it was not affected in the OFT or pharyngeal arch core mesoderm (Figure 1). 3D reconstruction analysis of section images provides an overall view of the expression domains affected by loss of *Tbx1* (Figure 1K–1P). Similar results were obtained at later stages (E9.0–11.5, not shown).

To confirm that *Wnt5a* is expressed in cardiac progenitors, we carried out qRT-PCR on the P19CL6 mouse embryonal carcinoma cell line that is able to differentiate into cardiomyocyte progenitors upon 5-azacytidine and/or DMSO treatment [11]. We found that *Wnt5a* is robustly expressed after 3 days of differentiation (Figure S1). The activation of *Wnt5a* expression roughly coincides with the up-regulation of *Tbx1* expression, suggesting that Tbx1 may have a role in the activation of *Wnt5a* rather than in its expression maintenance. We also found that loss of *Wnt5a* in these cells reduces cell migration, as tested by a wound-healing assay (Figure S2). Next we tested if Tbx1 can regulate *Wnt5a* expression in these cells. For this, we transfected graded amounts of a *Tbx1*-expressing vector into P19CL6 undifferentiated cells and evaluated expression of the endogenous *Wnt5a* gene by qRT-PCR. Results revealed that *Wnt5a* expression responds positively to Tbx1 in a dosage-dependent manner (Figure S3).

### *Tbx1* and *Wnt5a* interact genetically and their combined loss causes failure of SHF–derived structures

To test whether *Wnt5a* and *Tbx1* interact in vivo, we crossed *Tbx1* ^+/−^;*Wnt5a* ^+/−^ mice and determined the embryonic phenotype on the progeny at E18.5. Results of the analysis of 162 embryos are summarized in Tab. 1. Double heterozygous embryos (*Tbx1* ^+/−^;*Wnt5a* ^+/−^, n = 61) showed higher penetrance of the typical *Tbx1* haploinsufficiency phenotype (hypoplasia or aplasia of the 4th pharyngeal arch artery) than did the *Tbx1* ^+/−^ embryos (n = 17) (44.3% *vs.* 23.5%, p<0.05). However, we did not find any additional phenotypic abnormalities in double heterozygous embryos. We did not find any cardiovascular abnormality in *Wnt5a* ^+/−^ embryos (n = 30) consistent with previously reported data [6].

**Table 1:** Summary of phenotyping results of E18.5 embryos from *Tbx1* ^+/−^;*Wnt5a* ^+/−^ X *Tbx1* ^+/−^;*Wnt5a* ^+/−^ intercrosses.

E18.5	n	Normal(%)	Dead	AoAAbn	Ao-PTside byside + VSD	TAC + VSD
*WT*	**5**	5 (100)	-	-	-	-
*Tbx1+/−*	**17**	13 (76)	-	4 (23%)	-	-
*Wnt5a+/−*	**30**	30 (100)	-	-	-	-
*Tbx1+/−; Wnt5a+/−*	**61**	34 (56)	2	25 (41%)	-	-
*Tbx1+/−; Wnt5a −/−*	**22**	-	-	-	9	13
*Tbx1−/−*	**7**	-	-	-	-	7
*Wnt5a−/−*	**5**	-	-	-	5	-
*Tbx1−/−; Wnt5a+/−*	**15**	-	-	-	-	15
*Tbx1−/−; Wnt5a−/−*	**0**	-	-	-	-	-
**Total**	**162**					

AoA Abn: Abnormalities of the aortic arch and of the origin of the right subclavian artery.

Ao-PT: abnormal relative position of the aorta and pulmonary trunk, arranged side-by-side.

VSD: Ventricular septal defect, perimembranous.

TAC: Truncus arteriosus communis.

**Figure 2:**
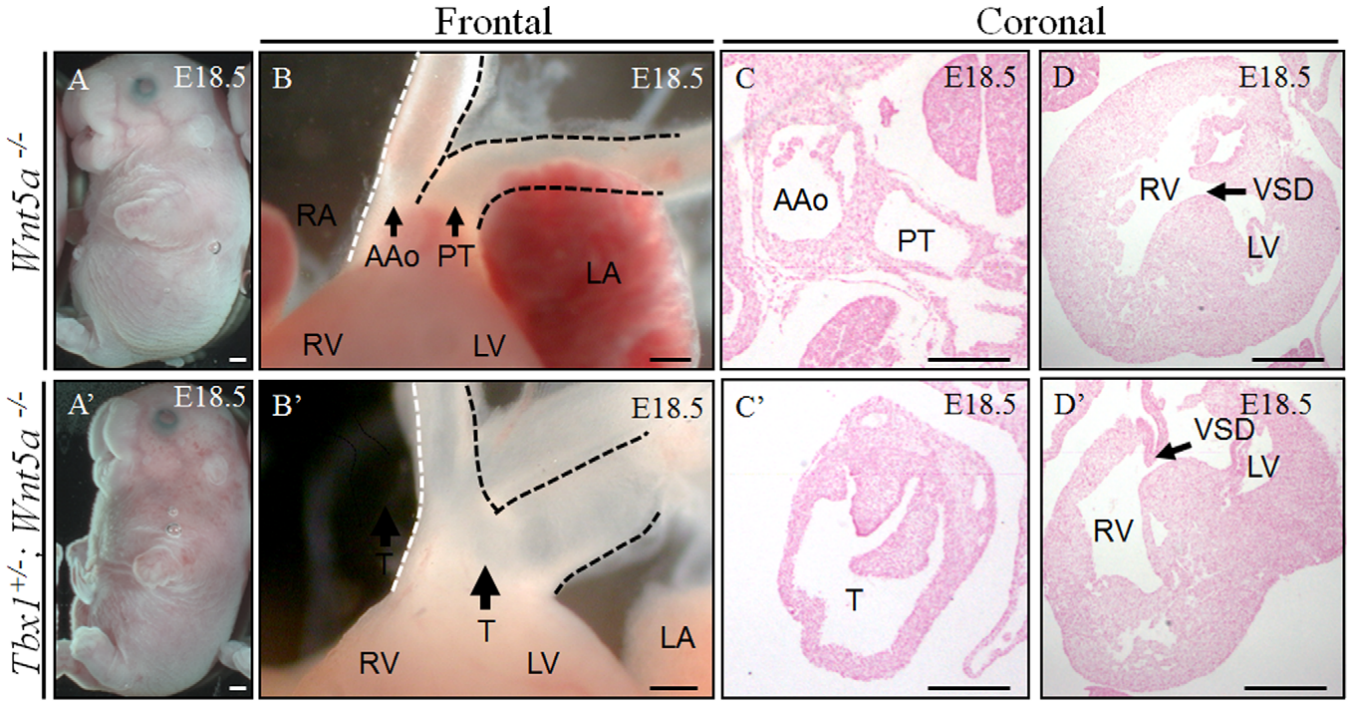
Tbx1 and Wnt5a interact genetically. (A–A′) Extracardiac phenotypes in *Wnt5a^−/−^* embryos included cleft palate, cleft lip, small thymus, truncated tail and truncated limbs at E18.5 (A, n = 5). In addition to the extracardiac phenotypes seen in *Wnt5a^−/−^* embryos, *Tbx1^+/^−*; *Wnt5a* ^−/−^ embryos had small ears and edema at E18.5 (A′, n = 22). (B–D′) Coronal sections of the heart revealed ventricular septal defects (VSD) and abnormal (side-by-side) positioning of the ascending aorta (AAo) and pulmonary trunk (PT) in *Wnt5a* ^−/−^ embryos (B–D, n = 5). 13 out of 22 (59%) *Tbx1^+/−^; Wnt5a^−/−^* embryos showed truncus arteriosus communis (TAC), which was not observed in the *Wnt5a* ^−/−^ littermate (B′–D′). T: truncus arteriosus communis. Scale bars: 1 mm in A–B′; 100 µm in C–D′.

Analysis of *Wnt5a* ^−/−^ embryos (n = 5) revealed ventricular septal defects (VSD) and abnormal positioning of the great arteries (aorta and pulmonary trunk), which were side-by-side (Figure 2B–2D). However, in none of these embryos did we observe truncus arteriosus communis (TAC), although this defect was previously reported for most *Wnt5a* ^−/−^ embryos [6]. This difference may be due to different genetic backgrounds. However, the extracardiac phenotype that we found was consistent with previously reported data (cleft palate, cleft lip, small thymus, truncated tail and limbs) (Figure 2A and data not shown). Analysis of *Tbx1* ^+/−^;*Wnt5a* ^−/−^ embryos (n = 22) revealed a more severe phenotype than in *Wnt5a* ^−/−^ embryos from the same crosses. In particular, out of 22 embryos examined, 13 (59%) showed additional abnormalities compared to *Wnt5a* ^−/−^ embryos, namely TAC, small ear and edema (Figure 2A′–2D′). The other embryos (9 out of 22 or 41%) were phenotypically indistinguishable from *Wnt5a* ^−/−^ embryos. Together, these results indicate a genetic interaction between *Tbx1* and *Wnt5a*.

**Figure 3:**
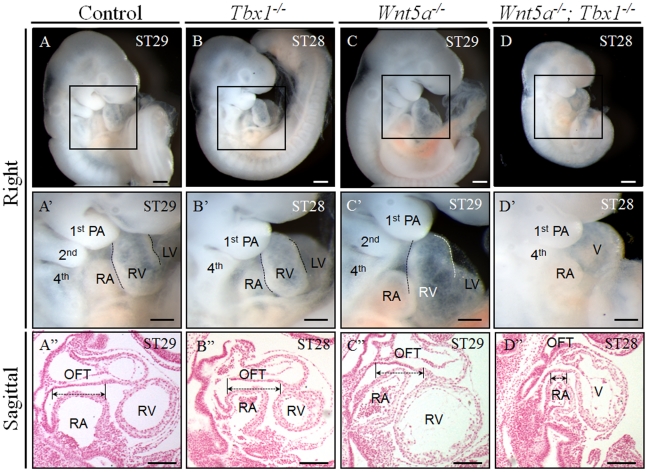
*Tbx1^−/−^;Wnt5a^−/−^* embryos have severe cardiac abnormalities. (A–D′) Whole mount histology study showed reduced overall size of *Tbx1^−/−^; Wnt5a^−/−^* embryos at E9.5 (n = 10), compared with control (WT), *Tbx1^−/−^* or *Wnt5a^−/−^* littermates. (A″–D″) Sagittal *sections of* these embryos revealed severe hypoplasia of the OFT and the right ventricle (RV) in the *Tbx1^−/−^; Wnt5a^−/−^* embryos. Scale bars: 100 µm.

**Figure 4:**
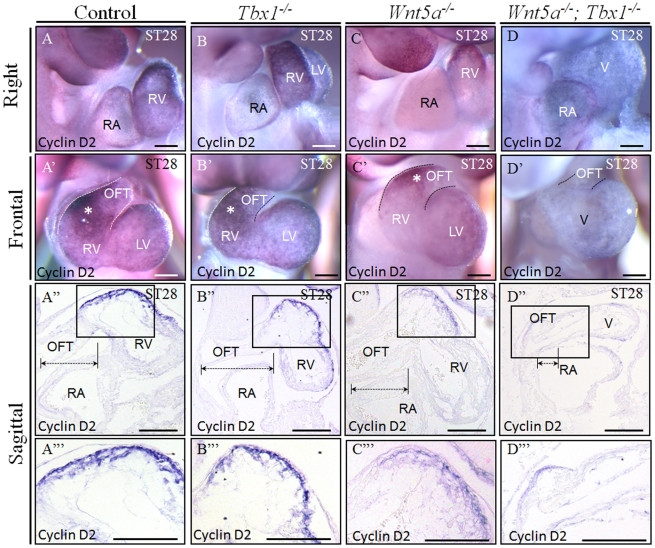
*Tbx1^−/−^;Wnt5a^−/−^* embryos have severe hypoplasia of SHF–derived heart. (A–D′) Right view (A–D) and frontal view (A′–D′) of *Tbx1^−/−^; Wnt5a^−/−^* embryos hybridized *in situ* with a cyclin D2 probe showed severe hypoplasia of the OFT and the RV at E9.5, compared with littermate controls. (A″–D″) Sagittal sections from whole-mount embryos hybridized with the cyclin D2 probe confirmed the severe cardiac defect in *Tbx1^−/−^; Wnt5a^−/−^* embryos. (A′″–D′″) high magnification of the regions identified with a square in panels A″–D″. Scale bars: 100 µm.

**Figure 5:**
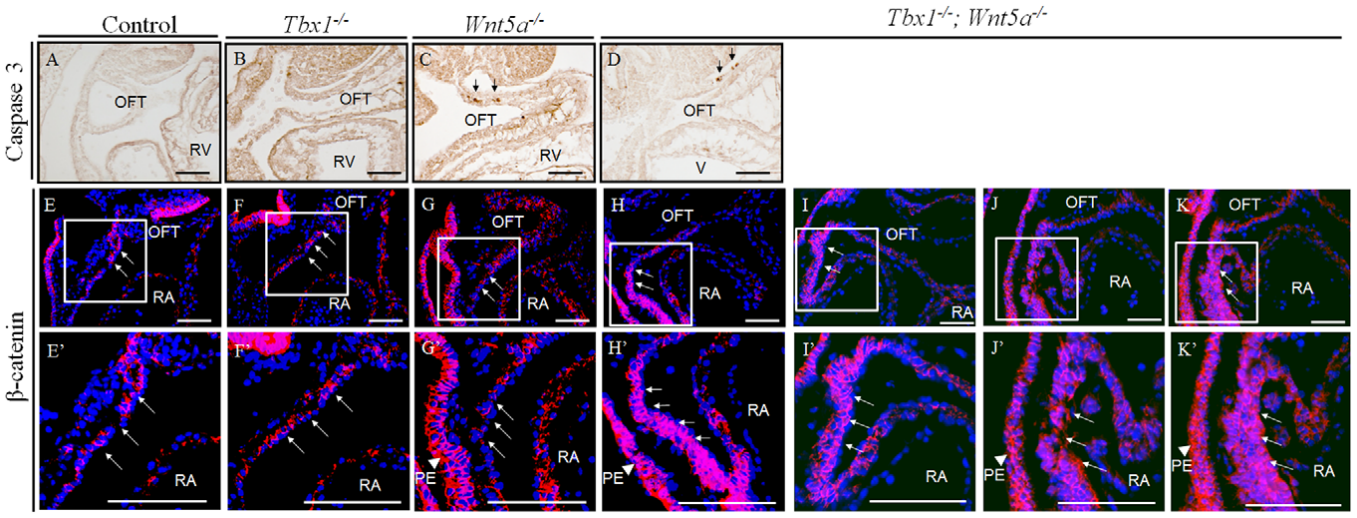
β-Catenin is upregulated in the SHF of *Tbx1^−/−^;Wnt5a^−/−^* embryos at E9.5. (A–D) Immunohistochemistry using an anti-cleaved Caspase 3 antibody showed no obvious difference in apoptosis between *Wnt5a* ^−/−^ and *Tbx1* ^−/−^; *Wnt5a* ^−/−^ embryos at E9.5. (E–K) Immunofluorescence using a β-catenin antibody showed no change of expression in the SHF (arrows) of *Tbx1* ^−/−^ or *Wnt5a* ^−/−^ embryos (E–G), while it was strongly upregulated in the SHF of *Tbx1* ^−/−^; *Wnt5a* ^−/−^ embryos at E9.5 (H–K panels from independent embryos). Squared regions are magnified in the bottom row of panels (E′–K′). Red: β-catenin; Blue: DAPI. PE: pharyngeal endoderm; OFT: outflow tract; RA: right atrium; Scale bars: 100 µm.

We did not retrieve any double homozygous embryos at E18.5 (n = 162), suggesting that this genotype is lethal during early embryogenesis. Therefore, we harvested embryos at earlier embryonic stages. The latest stage at which we found live *Tbx1* ^−/−^; *Wnt5a* ^−/−^ embryos was E9.5. At this stage, double homozygous embryos (n = 10) showed a severe cardiac phenotype characterized by severe hypoplasia of the OFT and right ventricle (RV), structures that sometimes appeared to be absent by visual inspection (Figure 3). Histological sections confirmed that the development of these structures is severely affected (Figure 3A″–3D″). We carried out in situ hybridization with a probe for *CyclinD2*, a marker of the proximal OFT and the right ventricle (RV) [12]. Results showed that *CyclinD2* was normally expressed in control, *Tbx1* ^−/−^ and *Wnt5a* ^−/−^ E9.5 embryos, but it was greatly reduced or undetectable in *Tbx1* ^−/−^;*Wnt5a* ^−/−^ embryos (Figure 4), confirming the severe hypoplasia of the OFT and of the RV. We asked whether the hypoplasia of these structures could be due to increased apoptosis. We carried out immunohistochemistry on E9.5 embryos using an anti-cleaved Caspase 3 antibody. Results showed very few positive cells in the OFT and RV of WT and *Tbx1* ^−/−^ E9.5 embryos, while both *Wnt5a* ^−/−^ and *Tbx1* ^−/−^;*Wnt5a* ^−/−^ embryos exhibited an increased number of apoptotic cells (Figure 5A–5D). However, we found no obvious difference in apoptosis between *Wnt5a* ^−/−^ and *Tbx1* ^−/−^;*Wnt5a* ^−/−^ embryos. Therefore, it is unlikely that apoptosis explains the severe heart phenotype of double mutants.

It has been reported that there is antagonism between the non-canonical and canonical Wnt pathways (e.g. [13], [14]). Therefore, we asked whether there could be alterations of β-catenin expression in our mutants. By immunofluorescence, β-catenin expression in the SHF did not change in *Tbx1* ^−/−^ or *Wnt5a* ^−/−^ embryos at E9.5 (Figure 5F–5G and 5F′–5G′), but it was upregulated in the SHF of *Tbx1* ^−/−^;*Wnt5a* ^−/−^ embryos (Figure 5H–5K and 5H′–5K′).

### *Wnt5a* is a target of Tbx1

**Figure 6:**
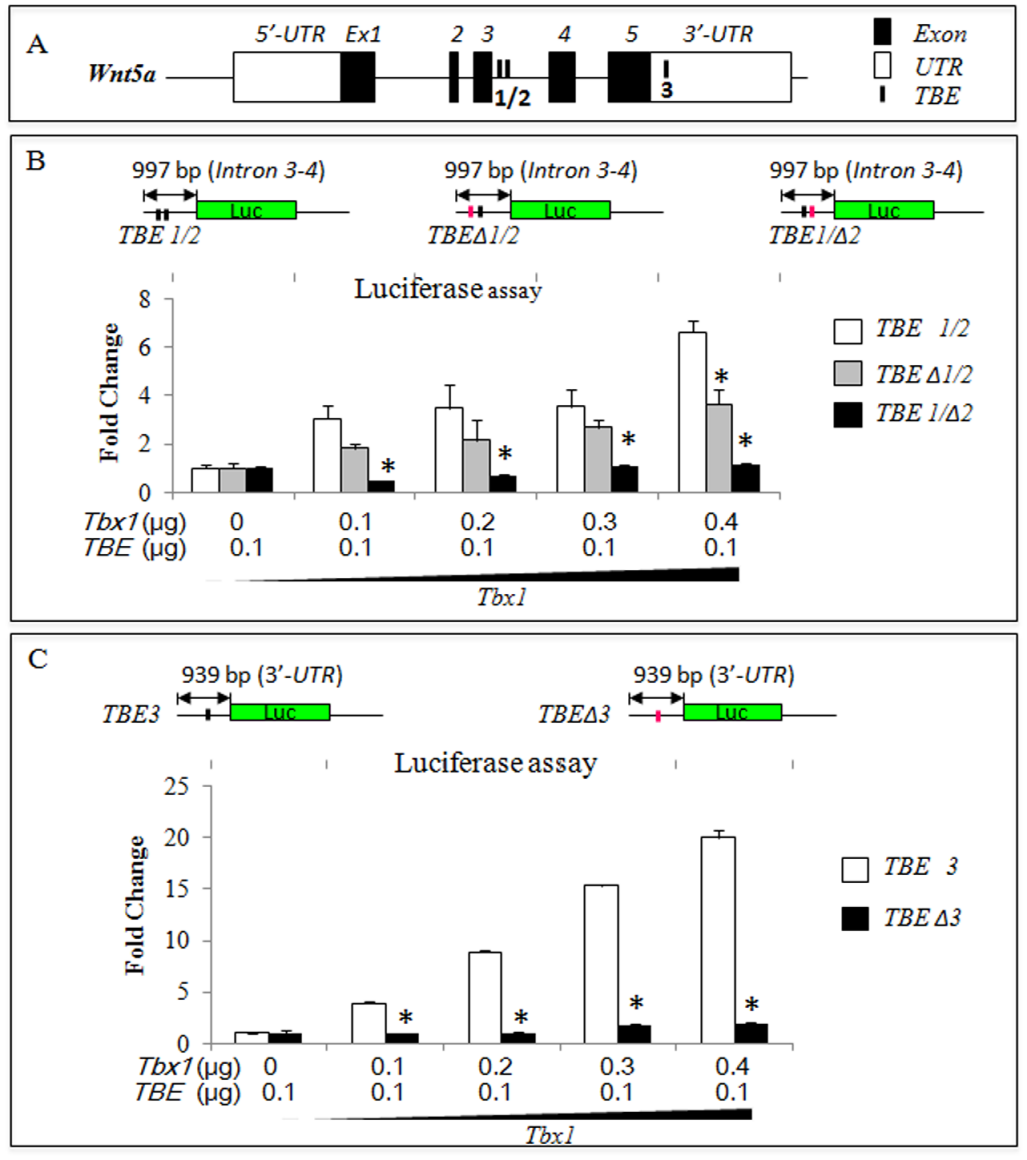
*Wnt5a* has conserved T-box binding elements (TBEs) that are required for response to *Tbx1* in luciferase assays. (A) Schematic illustration of three conserved TBEs of the mouse *Wnt5a* gene. (B–C) Mutation of the TBEs, indicated as TBE Δ1, Δ2, Δ3, individually or combined, significantly reduced the ability of Tbx1 to activate the *Wnt5a* luciferase reporters in JEG3 cells. Note that the TBE Δ1 mutation has a relatively mild effect but still significant. Asterisks indicate statistically significant difference (P-value less than 0.05) between the WT value and the Mutant value for each *Tbx1* dosage.

To understand whether *Wnt5a* may be a direct target of Tbx1, we searched for T-Box binding elements (TBEs) in a 20 kbp genomic sequence encompassing the mouse *Wnt5a* gene. We found three putative TBEs, two of which closely located in intron 3–4 (TBE1: AAGGGGTGAA, TBE2: GTAGGTGCCAGG) and one in the 3′-UTR (TBE3: AGAGGTGTTGCA) (Figure 6A). We next cloned two evolutionarily conserved (in human and mouse) DNA segments containing TBE1–2 and TBE3, into a luciferase reporter plasmid with a basic promoter (Figure 6B–6C). We also generated mutagenized constructs in which the three TBEs were mutated (the core sequence GTG was changed into AAA). We then carried out luciferase assays. Results showed that the two DNA segments responded well to transfected *Tbx1* (Figure 6B–6C). However, mutation of the TBEs, individually or combined, all had a significant impact on the ability of Tbx1 to activate the constructs (Figure 6B–6C), although the mutation of TBE1 had a relatively lower effect. Thus, these TBEs are required for Tbx1-induced activation in this assay.

**Figure 7:**
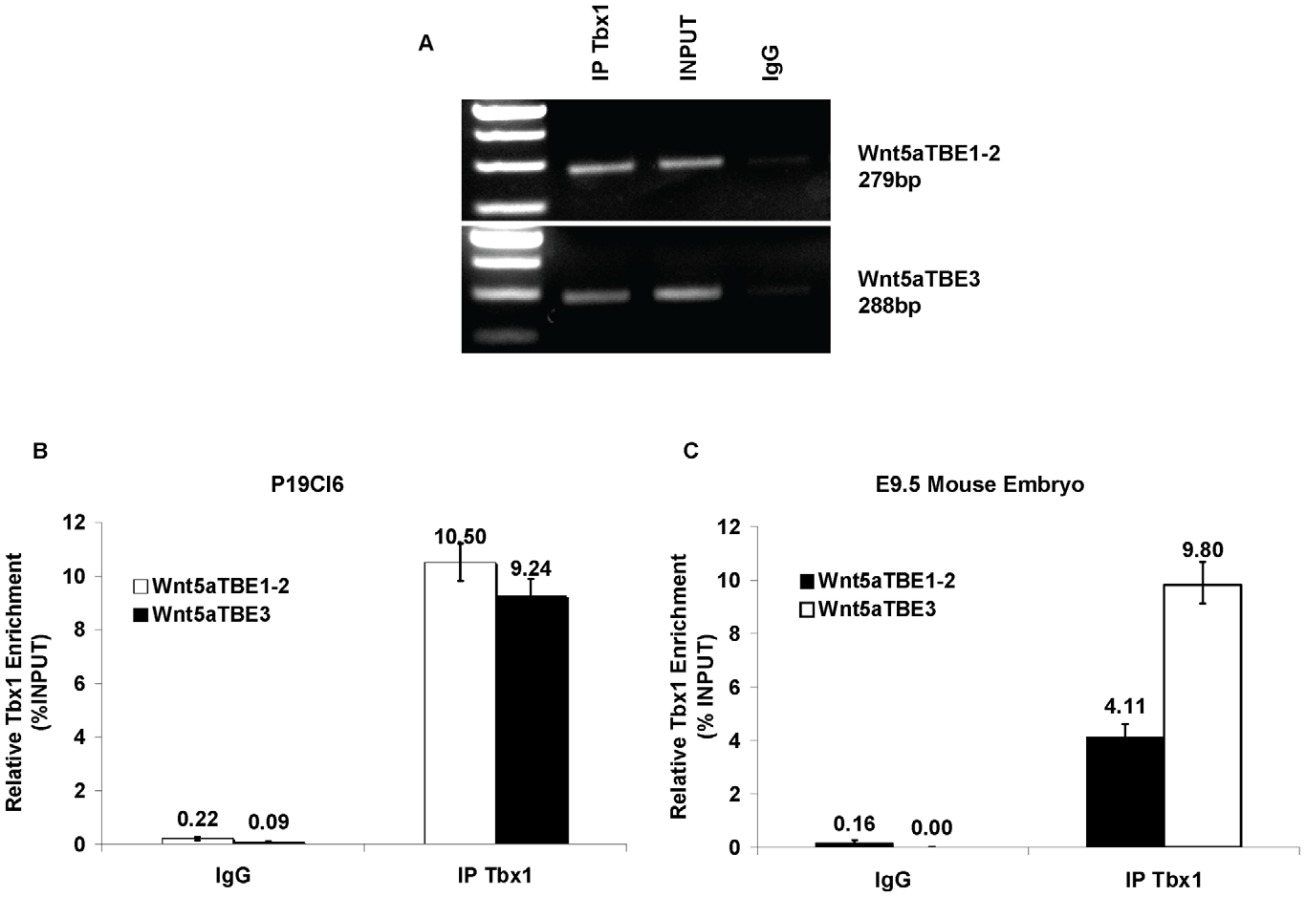
*Tbx1* occupies the TBEs of *Wnt5a* in P19Cl6 cells. Chromatin immunoprecipitation (ChIP) assays of P19Cl6 using an anti-Tbx1 antibody. (A) Standard PCR results using primers for the TBE1–2 and the TBE3 regions of *Wnt5a*. Amplification is clearly visible from the Tbx1-immunoprecipitated material and from the input sample but not from the IgG-immunoprecipitated material (negative control). (B) Quantitative ChIP assay (q-ChIP) on a similar experiment as the one shown in (A) but followed by quantitative real-time PCR. (C) Q-ChIP assay of E9.5 mouse embryos using an anti-Tbx1 antibody. The enrichment of TBE1–2 and TBE3 regions is evaluated compared to an internal control, which is a primer pair amplifying an ORF-free segment of mouse chromosome 14, and expressed as percentage of input. Values are from 3 experiments (mean±S.D.).

Next, we tested whether the endogenous Tbx1 protein occupies these TBE sites in P19Cl6 cells by standard and quantitative chromatin immunoprecipitation (ChIP) assays using a Tbx1 antibody. TBE1 and TBE2 were assayed together because they are too close to be assayed independently. Results demonstrated that indeed these sites are occupied by endogenous Tbx1 in P19CL6 cells (Figure 7A–7B). Next, we repeated the same assay using chromatin from E9.5 wild type embryos and again we could demonstrate enrichment at the *Wnt5a* TBE loci (Figure 7C).

### Tbx1 interacts with the BAF chromatin remodeling complex and with the Setd7 histone methyltransferase

**Figure 8:**
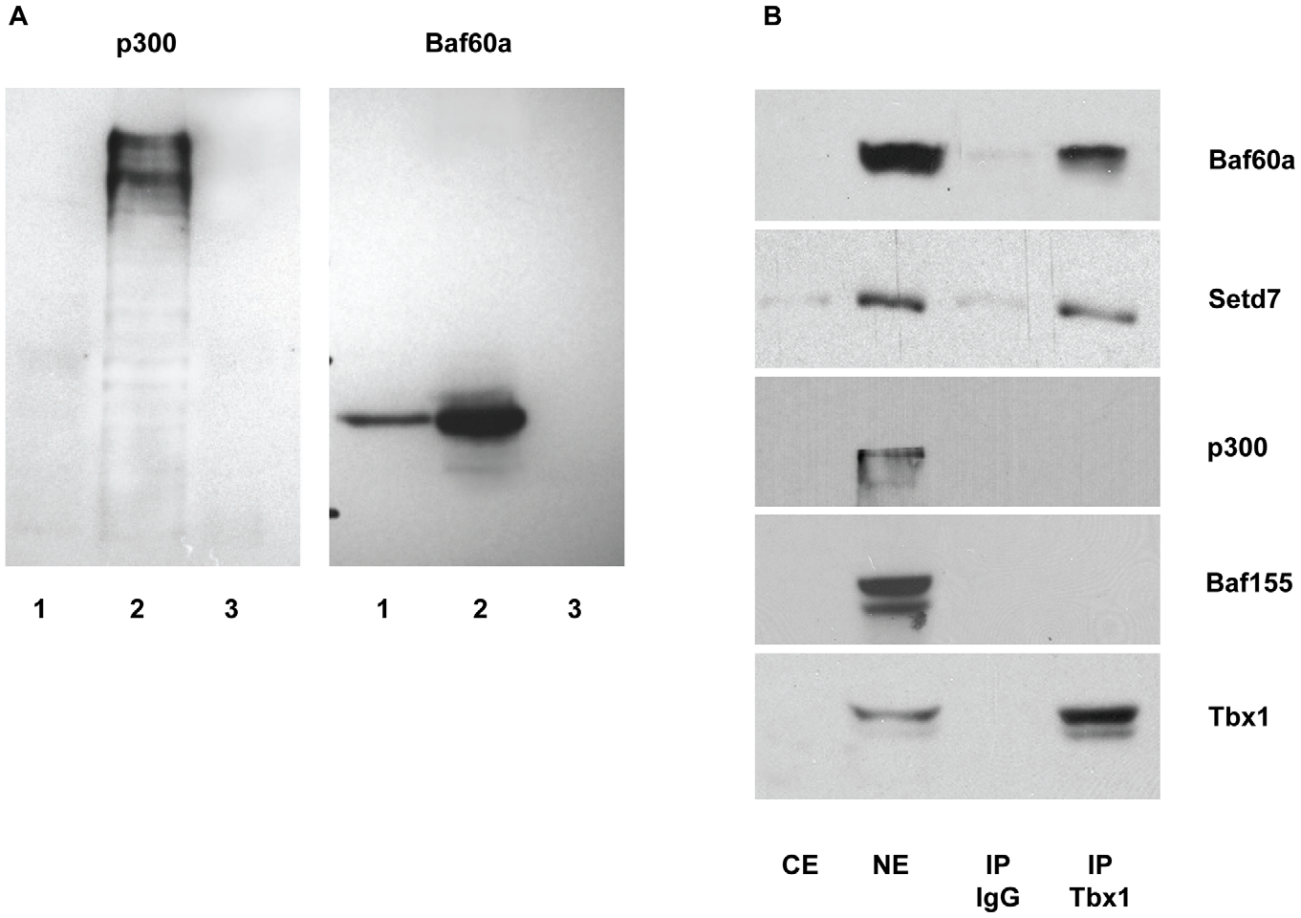
Tbx1 interacts with Baf60a and with Setd7, but not with p300 or Baf155. (A) Western blot analysis of affinity-purified nuclear extracts and controls. For both panels, Lane 1: Affinity-purified material from P19-Tbx1-PA cells (constitutively expressing a Tbx1-TEV-ProteinA construct); lane 2: nuclear extracts from the same cells; lane 3: affinity purified nuclear extracts from P19-PA cells (constitutively expressing a TEV-Protein A construct as a negative control). Note that p300 is not retained in the affinity purified material (left panel, lane 1), while Baf60a is retained (right panel, lane 1). (B) Western blot analyses of coimmunoprecipitation experiments with endogenous proteins from P19Cl6 cells. Immunoprecipitation was carried out using an anti-Tbx1 antibody or rabbit IgG (control). Western blots were carried out using anti-Baf60a, anti-Setd7, anti-p300, anti-Baf155 and anti-Tbx1 antibodies. CE: cytoplasmic extract (10% of input), NE: nuclear extracts (10% input).

**Figure 9:**
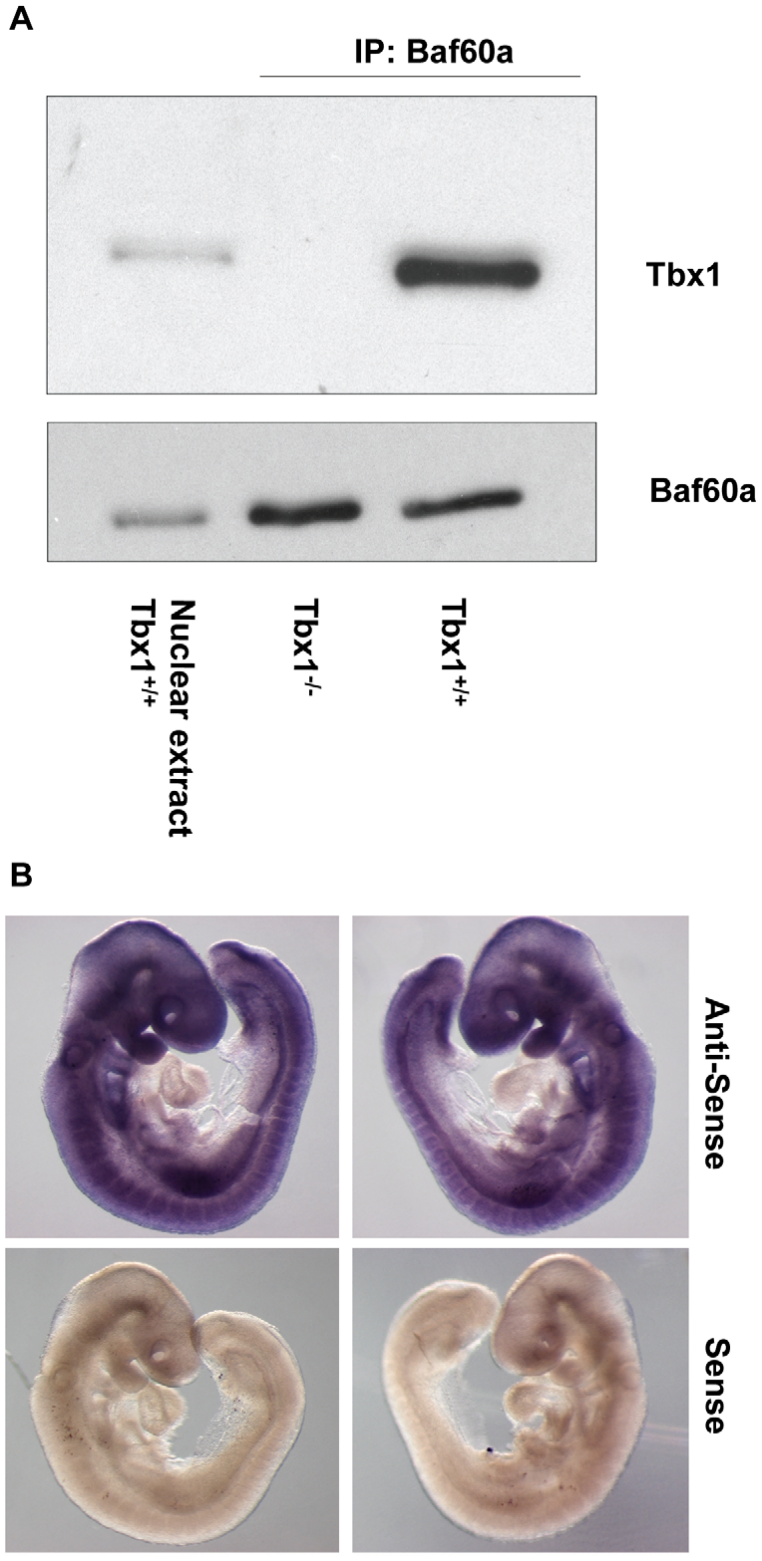
Tbx1 interacts with Baf60a in mouse embryo tissues. (A) Co-IP experiment showing interaction of the Tbx1 and Baf60a endogenous proteins in embryo tissues. The first lane on the left contains nuclear extracts from E9.5 WT embryos. The second and third lanes contains nuclear extracts from E9.5 *Tbx1* ^−/−^ and WT embryos immunoprecipitated with an anti-Baf60a antibody. Western blot with an anti-Tbx1 antibody clearly show co-immunoprecipitation in the WT sample. (B) Whole mount in situ hybridization with an anti-sense and sense (control) *Baf60a* probe. Note a nearly ubiquitous hybridization signal but with very little or no expression in the heart.

To begin to dissect the transcriptional machinery within which Tbx1 exerts its transcriptional functions, we tested by co-immunoprecipitation (co-IP) or affinity purification chromatin remodeling and histone modification proteins, Baf60a, Baf60c, Baf155, Setd7 and p300. Under the experimental conditions tested, we found that Baf60a and Setd7, but not Baf60c, Baf155 and p300, co-immunoprecipitated with Tbx1 (Figure 8 and Figure S4A)). The Baf60a-Tbx1 interaction was also confirmed using a GST pull down assay (Figure S4B). Furthermore, we confirmed Baf60a-Tbx1 co-IP using E9.5 embryo tissues (Figure 9A). At this stage, the *Baf60a* gene is expressed broadly, including the SHF, but is very weakly expressed or absent in the heart (Figure 9B). In contrast, Baf60c, an alternative component of the BAF complex, is mainly expressed in the heart and somites, and it has been previously shown to interact with another T-box transcription factor, Tbx5 [15]. While Tbx1 is mainly required in cardiac progenitors, where it is important to keep cells proliferating and to inhibit their differentiation, Tbx5 is important for cardiomyocyte differentiation. We compared expression of *Tbx1*, *Baf60a* and *Baf60c* mRNA during P19Cl6 cell differentiation. Results showed that *Tbx1* is expressed in the early phases of differentiation. *Baf60a* is particularly strong in these early phases and then its expression is reduced (although still robustly expressed). In contrast, *Baf60c* expression is very low in the early phases of differentiation and becomes quite strongly expressed as differentiation proceeds (Figure 1).

**Figure 10:**
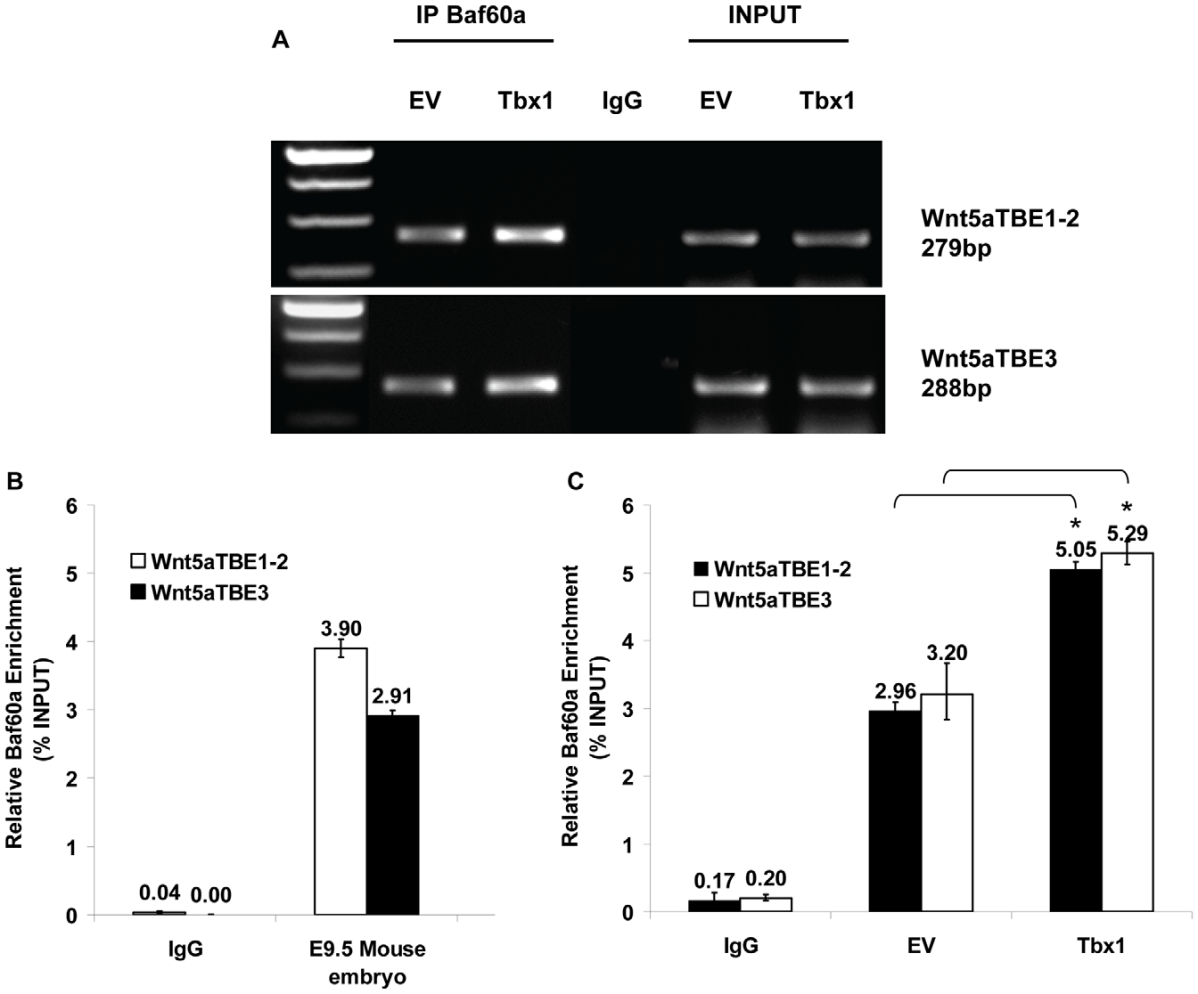
Baf60a occupies TBE regions of Wnt5a in P19Cl6 cells and embryo tissues. ChIP assays of P19Cl6 cells using anti-Baf60a antibodies or mouse IgG. (A) Standard PCR results clearly show amplification from the Baf60a-immunoprecipitated material and from the input sample but not from the IgG-immunoprecipitated material (negative control). (B) q-ChIP assay of E9.5 mouse embryos using an anti-Baf60a antibody. The enrichment of TBE1–2 and TBE3 regions is evaluated compared to an internal control. (C) q-ChIP assay on a similar experiment as the one shown in (A) but from P19Cl6 cells transfected with an empty vector (EV) or with a vector over-expressing Tbx1 (Tbx1), followed by quantitative real-time PCR. The immunoprecipitation was carried out with an anti-Baf60a antibody. Note that the TBE regions are significantly more enriched in the Tbx1 overexpressing sample, indicating positive correlation between Tbx1 dosage and Baf60a occupation. Values are from 3 experiments (mean±S.D.). Asterisks indicate statistically significant difference (P-value less than 0.05).

If the Baf60a-Tbx1 interaction were a feature of the transcriptional machinery at target genes, we would expect Baf60a to occupy the regions of the *Wnt5a* gene harboring the TBEs. To test this, we used ChIP with a Baf60a antibody on P19CL6 cells. Results demonstrated that indeed this protein occupies the TBE1/2 region as well as the TBE3 region (Figure 10A). The same results were obtained with a ChIP assay using chromatin from E9.5 embryos (Figure 10B).

### Tbx1 affects the chromatin status of TBE regions of *Wnt5a* and requires Baf60a for its function

**Figure 11:**
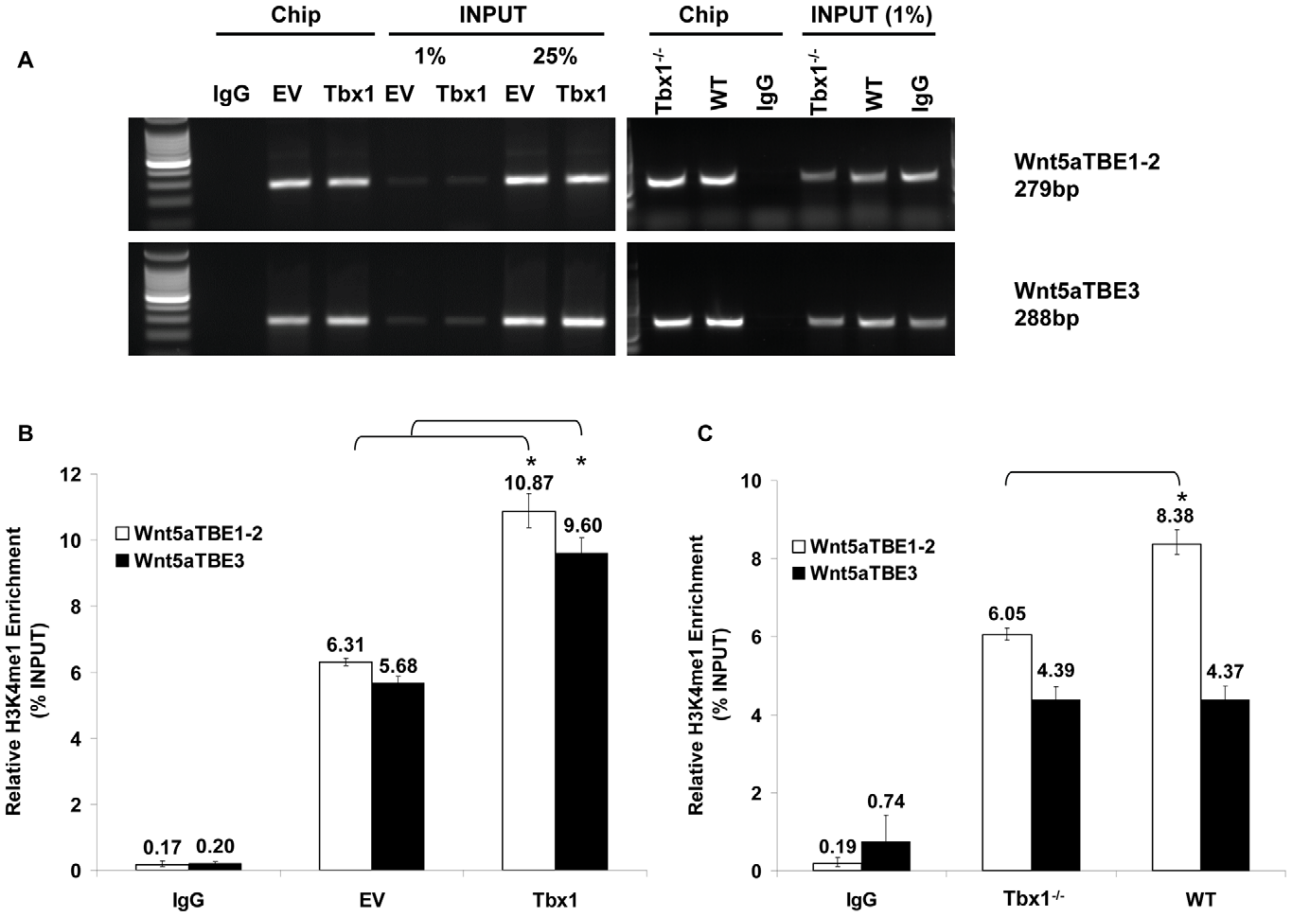
Tbx1 enhances H3K4 monomethylation of TBE regions of *Wnt5a* in cultured cells and in vivo. (A) ChIP assays of P19Cl6 cells using an anti-H3K4me1 antibody or mouse IgG. DNA isolated from immunoprecipitated material was amplified by standard PCR with primers to amplify the TBE1–2 and TBE3 regions of *Wnt5a*. The amplified PCR fragments were analyzed on 2% agarose gel. (B) q-ChIP assay from P19Cl6 cells transfected with an empty vector (EV) or with a vector over-expressing Tbx1 (Tbx1), followed by quantitative real-time PCR. The immunoprecipitation was carried out with an anti-H3K4me1 antibody. The enrichment of TBE1–2 and TBE3 regions is evaluated compared to an internal control. TBE regions are significantly more enriched in the Tbx1 overexpressing sample, indicating that increased dosage of Tbx1 correlates with increased methylation of H3K4 cross-linked to these DNA sequences. (C) q-ChIP assay from E9.5 *Tbx1^−/−^* and WT mouse embryos followed by quantitative real-time PCR. The immunoprecipitation was carried out with an anti-H3K4me1 antibody. The enrichment of TBE1–2 and TBE3 regions is evaluated compared to an internal control and expressed as percentage of input. Note the reduced enrichment of H3K4me1 at the *Wnt5a* TBE1–2 in *Tbx1^−/−^* embryos compared to WT. No change could be detected at the TBE3 locus. Values are from 3 experiments (mean±S.D.). Asterisks indicate statistically significant difference (P-value less than 0.05).

Given the interactions with the BAF complex and with Setd7, we postulated that Tbx1 would recruit Baf60a to the target gene and would affect histone methylation. Indeed, quantitative ChIP using a Baf60a antibody showed that Tbx1 increases significantly the enrichment of *Wnt5a* TBE sequences (Figure 10C). Thus, increased dosage of Tbx1 is sufficient to enrich the *Wnt5a* gene with a chromatin remodeling complex. Next, we asked whether Tbx1 dosage can modulate H3K4me1 at TBE regions of the *Wnt5a* gene. To this end, we carried out quantitative ChIP with an anti H3K4me1 antibody (Setd7 is a monomethyltransferase) on P19Cl6 cells transfected with *Tbx1*. Results indicated that there is indeed enrichment of H3K4me1 after increased dosage of Tbx1 (Figure 11A–11B). Next, we tested whether H3K4me1 is also affected by loss of *Tbx1* in vivo. To this end, we carried out qChIP assays using chromatin from WT and *Tbx1* ^−/−^ E9.5 embryos. Results indicated that there is a significantly higher enrichment of H3K4me1 in WT embryos compared to mutant embryos, but limited to the TBE1/2 locus (Figure 11C).

**Figure 12:**
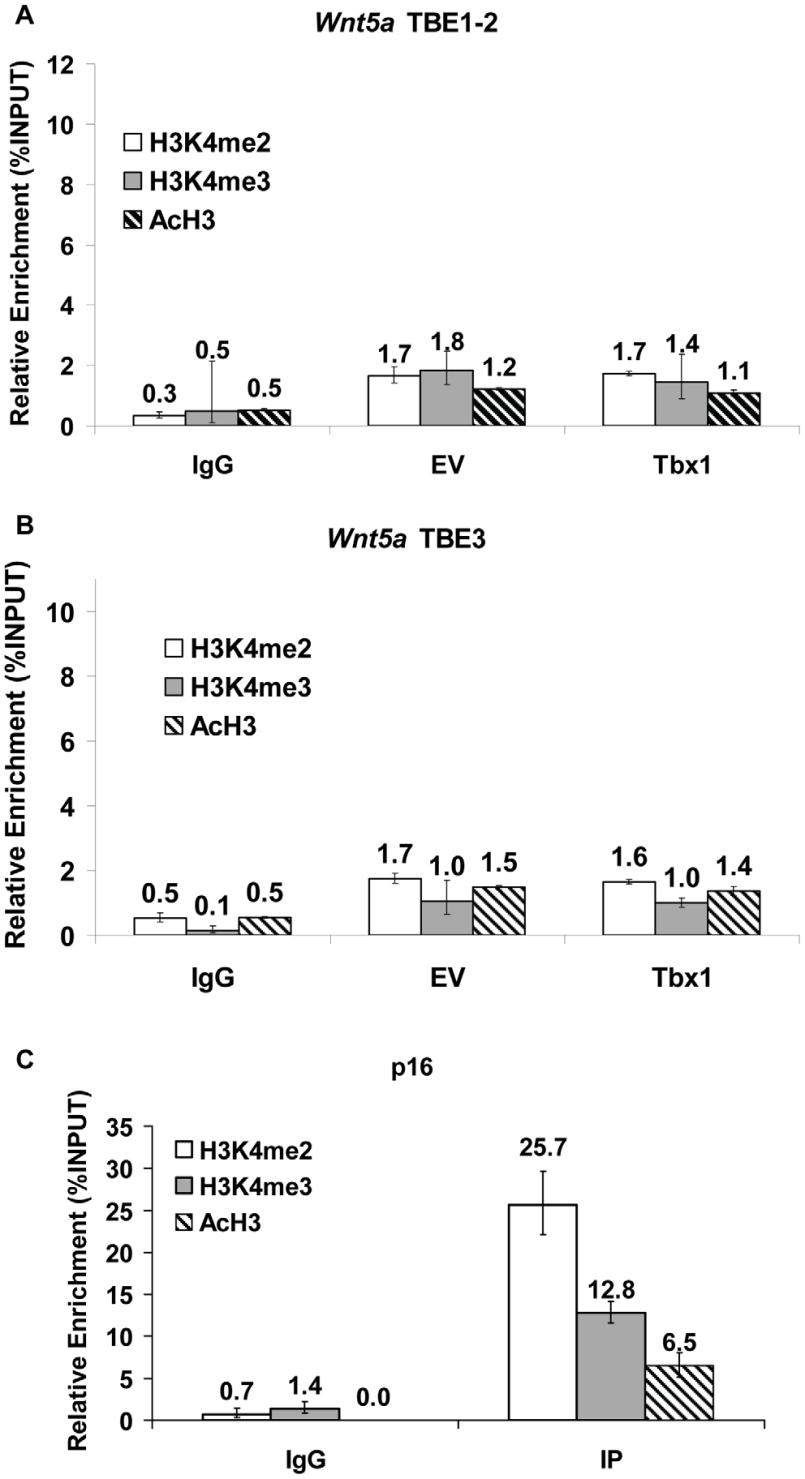
Effects of Tbx1 on H3K4 di- and tri-methylation and H3 acethylation status of the TBE regions of *Wnt5a*. q-ChIP assay from P19Cl6 cells transfected with an empty vector (EV) or with a vector over-expressing Tbx1 (Tbx1) followed by quantitative real-time PCR. The immunoprecipitation was carried out with anti-H3K4me2, anti-H3K4me3 or anti-AcH3 antibodies. (A) TBE1/2 region, (B) TBE3 region, and (C) *p16* promoter region (positive control). There is no enrichment for any of these histone modifications at the TBE regions. Values are from 3 experiments (mean±S.D.).

Next, we tested additional histone H3 modifications associated with gene activation. In particular, we carried out qChIP using chromatin from P19Cl6 cells transfected with an empty vector or with a vector over-expressing Tbx1 using antibodies against H3K4me2, H3K4me3, or AcH3 (recognizing acetylation of histone 3). Results indicated that none of these modifications are enriched in correspondence of the TBE loci of the Wnt5a gene, regardless of Tbx1 transfection (Figure 12).

**Figure 13:**
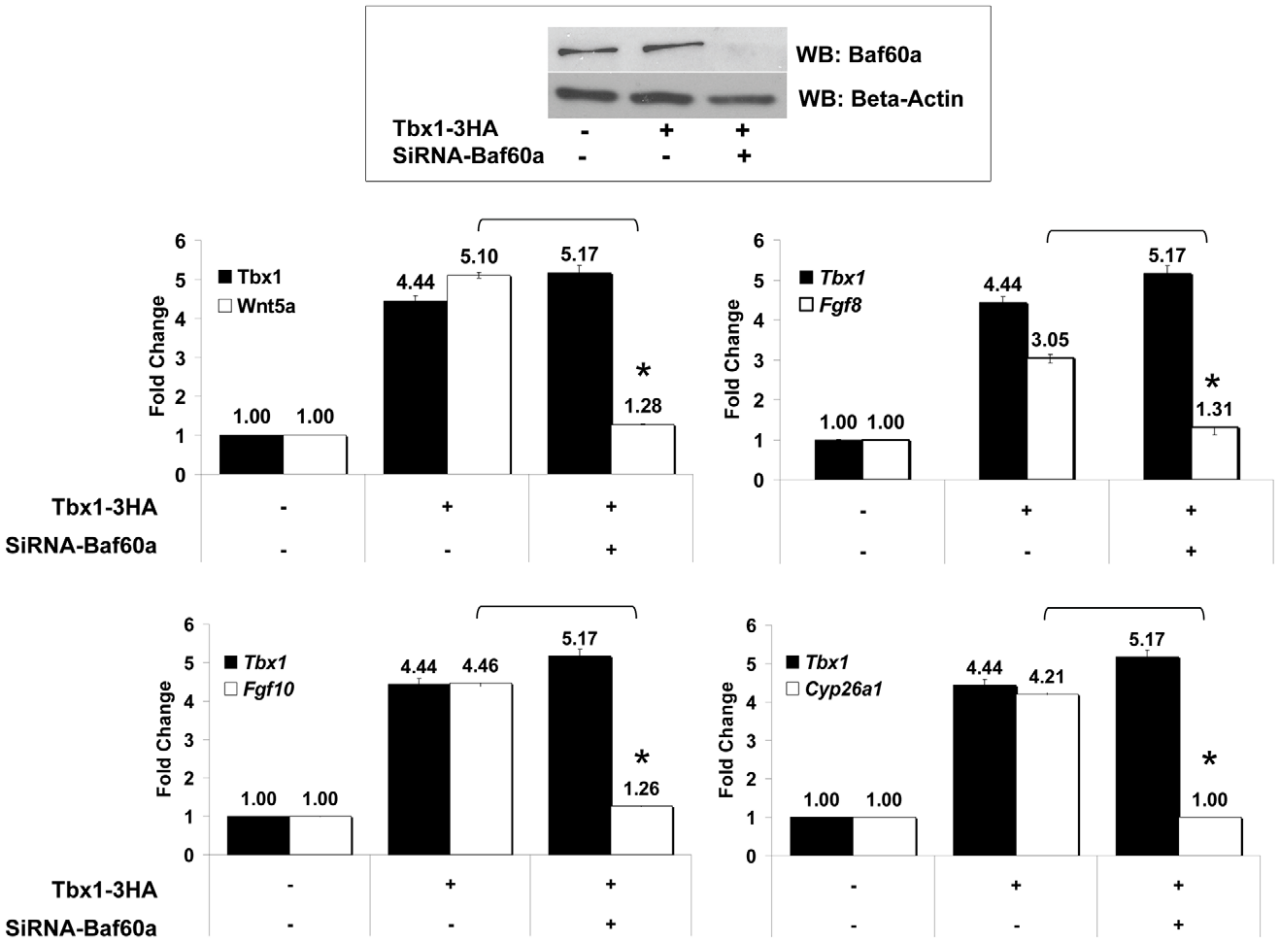
Tbx1 requires Baf60a to regulate target genes. P19Cl6 cells were transfected with empty vector or Tbx1-expression vector (Tbx1-3HA) and an anti-Baf60a siRNA or control siRNA. The western blot shown on the top panel shows the efficiency of the knock-down experiment. The histograms represent quantitative real-time PCR evaluation of the expression of Tbx1 (in black) and of 4 target genes at the experimental points indicated. In all cases, Baf60a knock-down abolishes the Tbx1-driven upregulation of these target genes. Values are from 3 experiments (mean±S.D.). Asterisks indicate statistically significant difference (P-value less than 0.05).

Finally, we asked whether Baf60a is important for Tbx1-induced upregulation of the *Wnt5a* gene. To this end, we knocked-down *Baf60a* expression by RNA interference and determined the ability of Tbx1 to regulate *Wnt5a* in the presence of reduced Baf60a dosage in P19CL6 cells. Results showed that after a knock down of approx. 80% of Baf60a, Tbx1 was unable to regulate *Wnt5a* (Figure 13). Interestingly, without Baf60a, Tbx1 was not able to activate other candidate targets (*Fgf8*, *Fgf10* and *Cyp26a1*) indicating that Baf60a is required for Tbx1 transcriptional activity in this context. Baf60a knock-down *per se* did not affect significantly the basal expression of endogenous *Tbx1*, *Wnt5a*, *Fgf8*, *Fgf10* and *Cyp26a1* in control experiments (Figure S5).

## Discussion

This work identifies *Wnt5a* as a novel and important transcriptional target of Tbx1. Combined loss of the two genes caused severe hypoplasia of SHF-derived heart segments, and early embryonic lethality. This is a much more severe phenotype than that caused by loss of the individual genes. The exact cause of the dramatic heart phenotype will require further investigation. However, our data suggest at least two possible mechanisms. Loss of *Tbx1* impairs the ability to expand the heart progenitors pool of the SHF [16], the loss of *Wnt5a* impairs their ability to migrate (and/or to be correctly orientated) into the heart. Thus, a double loss would essentially amount to a double hit upon cardiac progenitors, which would be fewer in number and less capable of contributing to the heart. Another possible cause of the severe phenotype might be an interference with the Wnt canonical signaling pathway in the double mutants, as suggested by the observed upregulation of β-catenin expression. Constitutive activation of Wnt canonical signaling in the SHF leads to severe heart abnormalities that are similar to those observed in the double homozygous mutants [17].

The importance of the non-canonical Wnt signaling for OFT development has already been illustrated by the study of *Wnt11* mouse mutants [18]. *Wnt5a* and *Wnt11* are both expressed in the OFT but, in contrast to *Wnt5a*, *Wnt11* is not expressed in the SHF. It would be interesting to cross the two mutants to see whether there is functional redundancy in the OFT proper.

Because of the clear in vivo importance of the Tbx1->*Wnt5a* transcriptional control for SHF function, we decided to focus our attention on the mechanisms regulating this control, as a possible paradigm for transcriptional control in the SHF.

We found an interaction between Tbx1 and Baf60a. The latter is a component of the BAF (Brg1-Brm Associated Factor) complex, which is related to the yeast and fly SWI-SNF chromatin remodeling complex. The canonical function of the BAF complex is to utilize its ATPase activity to rearrange the nucleosome distribution of chromatin, thus playing a crucial role in regulating accessibility to components of the transcriptional machinery, and thereby gene expression, either positively or negatively [19]. One of the critical issues concerning the biology of the BAF complex, is how it is targeted to genes that need to be regulated, i.e. how is specificity achieved despite the apparent homogeneity of the core components of the complex. One possibility is that specificity is provided by non-core subunits and by the recruitment activity operated by transcription factors that target specific genes. Our ChIP data suggest that this may be the case, as Tbx1 appears to recruit a BAF subunit onto the *Wnt5a* gene. Interaction with a histone methyltransferase would further help transcription, for example by stabilizing the remodeling machinery on the locus. We show that indeed Tbx1 expression correlates with increased H3K4 monomethylation of the *Wnt5a* TBE loci in cultured cells and in vivo. Interestingly, Tbx1 co-immunoprecipitates with Baf60a but not Baf60c. These are two alternative subunits of the BAF complex, possibly associated with different target genes and different cellular differentiation states. Baf60a has been associated with undifferentiated/multipotent status [20], [21], while Baf60c has been associated with differentiating muscle cells (cardiac or skeletal) [22], [23]. We also show here that Baf60a tends to be downregulated during P19Cl6 cell differentiation, while Baf60c is upregulated. This finding is supported by in vivo expression data indicating that Baf60a is poorly represented in differentiated heart tissue in contrast to Baf60c, which is mostly expressed in heart and somite tissue [15]. Thus, it is possible that an exchange of BAF subunits plays a role in the passage from the progenitor state to the differentiated cardiomyocyte state. Exchange of BAF subunits has already been described during differentiation from neural progenitors to neurons (Baf53a to Baf53b, and Baf45a to Baf45b) [24].

Exactly what promoters or enhancers exchange BAF subunits during cardiac differentiation would be an interesting question to address using genome-wide ChIP-seq studies.

Setd7 is a H3K4 monomethyltransferase [25]–[27] already shown to interact with another T-box transcription factor named Tbx21 (also known as Tbet) [28]. It is thus tempting to speculate that interaction with the BAF complex and histone methyltransferases is a common feature of T-box proteins and at the core of the transcriptional function of these important transcription factors.

Interestingly, our tissue culture experiments evidentiated that Tbx1 has a positive effect on H3K4me1 enrichment, but not H3K4me2, H3K4me3, or H3 acetylation. This suggests that Tbx1 promotes de novo methylation H3K4 at TBE enhancers perhaps making them a target for additional regulators.

Overall, our data support a model by which Tbx1 regulates *Wnt5a* by interacting with and perhaps recruiting a specific subunit of the BAF complex, along with the histone modifier enzyme Setd7, resulting in activation or enhancement of transcription of the target gene. While a number of important molecular details remain to be clarified, our data using a tissue culture model indicate the importance of Baf60a for Tbx1-induced regulation of *Wnt5a* and perhaps other target genes. Hence it is reasonable to speculate that this BAF subunit is a key cofactor for Tbx1 function in cardiac progenitors.

## Materials and Methods

### Ethics statement

All animal experimentations were carried out according to animal welfare regulations and guidelines of the USA and of the European Union.

### Mouse lines

We used the mouse lines *Tbx1* ^+/lacZ^ (here cited as *Tbx1* ^+/−^) [29], available through the EMMA repository, and *Wnt5a* ^+/−^ [10] available through the Jackson Laboratories. Both are null alleles. Genotyping was carried out according to instructions provided by the original reports.

All crosses were carried out in conventional, clean facilities in a C57Bl6/129SvEv mixed genetic background.

### Mouse embryo phenotyping

Embryos were examined after manual dissection under a stereo microscope. In most cases we also carried histological sectioning of paraffin-embedded specimens. Whole mount in situ hybridization according to standard methods. Embryos were photographed and then sectioned. In some cases we used images of sections for 3D reconstruction using the Amira software.

### Constructs and cell lines

P19CL6 cells were grown in Dulbecco-Modified Minimal Essential Medium supplemented with 10% fetal bovine serum. For cardiomyocytes differentiation the cells were plated at a density of 5.0×10^5^ cells/well on a 35-mm tissue culture dish. The next day, when cells reached ∼90% confluence, the medium was replaced with a growth medium containing 10 µM 5-Azacytidine for 24 h [11]. After treatment with 5- Azacytidine, cells were incubated in the growth medium containing 1.0% DMSO that was changed daily in order to remove the cell debris resulting from cell death. The experimental days were numbered consecutively beginning from the day of treatment with 5- Azacytidine (day 0).

The generation of the stably transfected cell lines P19-Tbx1-TEV-PA and P19-TEV-PA has been described [30]. These cell lines have been used for affinity purification experiments (see below).

For transient transfection, cells were cultured in 10 cm dishes until 60–70% confluent and transfected with Fugene6 (Roche) following the manufacturer protocol. Dharmacon ON-TARGETplus SMARTpool Baf60a/Smarcd1 siRNA was used to knockdown Baf60a expression using Fugene6 transfection reagent. ON-TARGETplus siCONTROL Non-targeting pool was used for control transfections.

### Protein extraction

P19Cl6 cells pelleted cells were resuspended in CE buffer (10 mM HEPES, 60 mM KCl, 1 mM EDTA, 0.075% v/v NP40, 1 mM DTT and 1X protease inhibitors, pH 7.6), and centrifuged. The nuclei were washed with 5× pellet volumes of cold CE buffer without detergent and centrifuged. 2× pellet volume of NE buffer (20 mM TrisHCl, 420 mM NaCl, 1.5 mM MgCl2, 0.2 mM EDTA, 25% Glycerol and 1X protease inhibitors, pH 8.0) was added to the nuclear pellet and incubated on ice for 30 min. Nuclear and cytosolic extracts were recovered spinning at maximum speed for 30 min to pellet any nuclei.

### Wound healing assay

P19Cl6 cells were cultured to confluent monolayer in 12-well. Two hours before the experiment, we treated cells with mitomycin c (10 µg/ml) and then we made a scratch wound using a standard 200-µl pipette tip. Wounded monolayers were washed with PBS and digitally photographed for the 0 hour timepoint using an inverted microscope equipped with a digital camera (Leica AF6000LX time-lapse). Images of the wound were acquired every 30 min for 24 h. Subsequently, pictures were analyzed using the “Image J” software. The wound healing effect was calculated as area wound closure compared with the area of the initial wound. Briefly, the distance between the wound margins was measured at 0 hours and again every 4 hours post-wounding for 24 h. 12 hours post–wounding the following formula was used to evaluate the area wound closure: distance^t = 12 h^-distance^t = 0 h^. Data represent the average of at least 3 independent experiments (2 wells/experiment).

### Affinity purification

Native affinity purification was performed with strains P19-Tbx1-PA and P19-PA as previously described [30], [31]. Briefly, nuclear extracts were transferred to Poly-Propylene Chromatography Columns with IgG sepharose beads (Amersham) to capture Tbx1-TEV-Protein A-containing complexes. Then, the columns were subjected to protease TEV cleavage to release the Tbx1-containing protein complexes, which were recovered and transferred into PVDF membrane (Amersham) for Western blotting analyses. The antibodies used for immunoblotting were the monoclonal anti-Baf60a antibody (BD Biosciences, #611728) and the anti-p300 (BD Biosciences, #554215).

### Co-immunoprecipitation

For co-immunoprecipitation experiments, nuclear extracts of P19Cl6 cells were quantified using a modified Bradford procedure (Bio-Rad Laboratories, Hercules, CA). Approximately 100 µg of nuclear extracts were incubated with an anti Tbx1 antibody (Abcam, #ab18530) or Baf60a antibody (BD Biosciences, #611728) or rabbit/mouse IgG (Santa Cruz Biotechnology, #2027) and then incubated with Protein A/G PLUS agarose (Santa Cruz Biotechnology) at 4°C ON. The samples were washed 6 times with IPP150 (10 mM Tris-HCl, pH 8.0; 150 mM NaCl; 0.1% NP-40) and resuspended in SDS sample buffer. 10 µg of nuclear extracts (10% Input) and immunoprecipitated samples were detected using Western blot analysis. We used anti-Baf60a antibody (BD Biosciences, #611728), an anti-Setd7 antibody (Abcam, #ab71214), anti-p300 (BD Biosciences, #554215) and anti-SMARCC1/Baf155 (Abcam, #ab72503).

### Chromatin immunoprecipitation

P19Cl6 cells were cross-linked using 1% formaldehyde at room temperature for 15 min, and the reaction was stopped using glycine at a final concentration of 0.125 M for 5 min. Cells were then lysed in 1 ml of lysis buffer (10 mM HEPES, 60 mM KCl, 1 mM EDTA, 0.075% v/v NP40, 1 mM DTT and 1X protease inhibitors, pH 7.6) on ice for 10 min, dounced using a 2 ml B dounce to release nuclei. Isolated nuclei were suspended in Nuclei lysis buffer (20 mM TrisHCl, 420 mM NaCl, 1.5 mM MgCl2, 0.2 mM EDTA, 25% Glycerol and 1X protease inhibitors, adjusted to pH 8.0), incubated on ice for 30 min, washed in nuclei lysis buffer and sonicated to obtain 200–500 bp. Sonicated chromatin was immunoprecipitated 10 µg of a Tbx1 antibody (Abcam, #ab18530), 2 µg of Baf60a antibody (BD Biosciences, #611728), 2 µg of H3K4me1 antibody (Abcam, #ab8895), 2 µg of Dimethyl-Histone H3 (Lys4) (Millipore, #07-030), 5 µg of Anti-trimethyl-Histone H3 (Lys4) (Millipore, #07-473), 5 µg of Anti-acetyl-Histone H3 (Millipore, #06-599), or normal rabbit/mouse IgG (Santa Cruz Biotechnology, #2027). Samples were then incubated with 20 µl of protein A/G PLUS agarose (Santa Cruz Biotechnology) at 4°C ON. The samples were extensively washed and incubated in an elution buffer (1% SDS and 0.1 M NaHCO3) at 30°C for 20 min. Cross-linking of protein-DNA complexes was reversed at 65°C ON, followed by treatment with DNase-fre RNase A for 30 min at 37°C and 100 µg/ml proteinase K for 2 h at 55°C. Phenol/chloroform-extracted, and ethanol-precipitated DNA was resuspended in 50 µl of H2O and subjected to PCR amplification (see below). ChIP experiments using embryo tissue was carried out as follows. WT and *Tbx1^−/−^* E9.5 mouse embryos were dissected, their heads were cut off and the trunks were stored at −80°C. Pools of 5 embryos were used for each ChIP experiment. Each ChIP experiment was performed using 5 µg of H3K4me1 (Abcam, #ab8895), 10 µg of TBX1 antibody (Abcam, #ab18530) and 5 µg or 10 µg of normal rabbit IgG (Santa Cruz Biotechnology, #2027) as negative control with the LowCell ChIP Kit (Diagenode). In brief, the embryos were fixed in 1% formaldehyde at room temperature for 15 min and then glycine was added to stop the reaction to a final concentration of 0.125 M for 5 min. Subsequently, embryos were washed with PBS, disaggregated by dounce homogenization in cold PBS and then resuspended in cell lysis buffer (5 mM PIPES pH 8.0, 85 mM KCl, 0.5% Igepal supplemented with protease inhibitors). Next, nuclei were resuspended in 130 µl of Buffer B (LowCell ChIP Kit reagent) and chromatin was sheared into 200–500 bp long fragments using the Covaris S2 Sample Preparation System (Duty Cycle: 5%, Cycles: 6, Intensity: 2, Temperature: 4°C, Cycles per Burst: 200, Power mode: Frequency Sweeping, Cycle Time: 60 seconds, Degassing mode: Continuous). Following steps included incubation of the sheared chromation with antibody coated beads over night, several washing steps and reverse crosslinking. Next, equal DNA amounts of input and immunoprecipitated DNA were initially used as a template for conventional PCR amplification of the TBE1–2 region of Wnt5a (Wnt5a TBE1–2_1_F 5′-CTTCCCCTGGTGTGGATATG-3′, Wnt5a TBE1–2_1_R 5′-AGAGGCTCCTTCCAGTCCTC-3′) and TBE3 region (wnt5a TBE-3_1_F 5′-ACTGCTGGTAGGGCAGAAAA-3′, wnt5a TBE-3_1_R 5′-TCAGGCACCATTAAACCACA-3′).

For quantitative ChIP, we next carried out real-time PCR of the immunoprecipitated DNA and inputs, using the FastStart Universal SYBR Green Master kit (Roche) and the 7900HT Fast Real-Time PCR System (Applied Biosystems). ChIP signals were normalized to that of an internal control amplimer that we have selected from an ORF-free region of mouse chromosome 14 (INT-XIV-1F 5′- TTCTTGTCCACAGCCCTCTT-3′, INT-XIV-1R 5′-TGGTGGAAGAGGAGACATCC-3′). PCR efficiencies were determined for each primer pair using standard curves. PCR was carried out on 2 µl of 1/100 dilution of the input or 2 µl of immunoprecipitated samples.

### Expression and purification of GST-Tbx1 fusion proteins

A GST-Tbx1 expression vector was kindly provided by Dr. Amendt (IBT, Texas A&M University, Houston, TX USA) and transformed into E. coli BL21(DE3) cells. Protein synthesis was induced by the addition of IPTG to a final concentration of 0.1 mM. GST pull-down assay was performed using ProFound Pull-Down GST Protein: Protein Interaction Kit (PIERCE).

### RNA extraction, cDNA synthesis, and Q-RT-PCR

RNA was extracted from P19Cl6 cells using TRI-Reagent (Ambion/Applied Biosystems) according to the manufacturer's protocol. Extracted RNA was treated with DNA-free Kit (Ambion/Applied Biosystems). cDNA was synthesized from 2 µg total RNA (normalized via UV spectroscopy) using the High Capacity cDNA Reverse Transcription Kit, according to the manufacturer's instructions (Applied Biosystems). Target cDNA levels were compared by Q-RT-PCR in 20-µl reactions containing 1× SYBR green (Applied Biosystems), 100 µm of each primer, and we used the 7900HT Fast Real-Time PCR System (Applied Biosystems). Results were normalized against glyceraldehyde-3-phosphate dehydrogenase (GAPDH) expression, unless otherwise indicated. Relative expression was evaluated using the delta-delta-cycle threshold method.

## Supporting Information

Figure S1 Expression analyses by quantitative real time PCR of genes *Tbx1*, *Wnt5a*, *Baf60a/Smarcd1* and *Baf60c/Smarcd3* in undifferentiated P19Cl6 cells (day 0) and during the first 5 days of treatment to induce differentiation.
(TIF)

Figure S2 In vitro, wound healing assay performed on P19Cl6 cells before and after Wnt5a knock-down by siRNA. (A) Western blot demonstrating knock-down of Wnt5a expression. (B) Microphotographs of the wound healing assay on P19Cl6 cells. (C) Summary of results expressed as area of closure with and without Wnt5a over time, up to 24 hours. Data were obtained by time-lapse microscopy. Control cells recover a significantly larger area after the wound (P = 0.01, t-test).
(TIF)

Figure S3 *Tbx1* and *Wnt5a* gene expression analysis by quantitative real time PCR assay in cells expressing *Tbx1* after transfection with the indicated amounts of a *Tbx1* expression vector. Each data point is expressed as the mean of three replicates ± SD.
(TIF)

Figure S4 (A) Western blot analyses of coimmunoprecipitation experiments using the anti-HA-immunoprecipitation kit. P19Cl6 cells were co-transfected transiently with Tbx1-3HA and Baf60c-Flag expression vectors). Western blots were carried out using anti-HA and anti-Flag antibodies. NE: nuclear extracts (10% input). (B) Direct interaction assay using GST pull-down shows that Tbx1 interacts with Baf60a. Western blot analysis of GST-Tbx1 pull-down from a lysate of P19Cl6 cells overexpressing Baf60a. The input lane shows Tbx1 expression in the lysate.
(TIF)

Figure S5 Quantitative real-time PCR evaluation of the expression of *Tbx1*, *Cyp26a1*, *Fgf8*, *Fgf10*, and *Wnt5a* with and without *Baf60a* knock-down by siRNA in undifferentiated P19Cl6 cells. Note that the knock-down of *Baf60a* has no significant effect on the expression of these genes (P-value more than 0.052.
(TIF)
