# Supplementary figures and images for: Transcriptional Control in Cardiac Progenitors: Tbx1 Interacts with the BAF Chromatin Remodeling Complex and Regulates Wnt5a
Source: PLoS Genet. 2012 Mar 15;8(3):e1002571. doi: 10.1371/journal.pgen.1002571 (PMC3305383; doi:10.1371/journal.pgen.1002571)

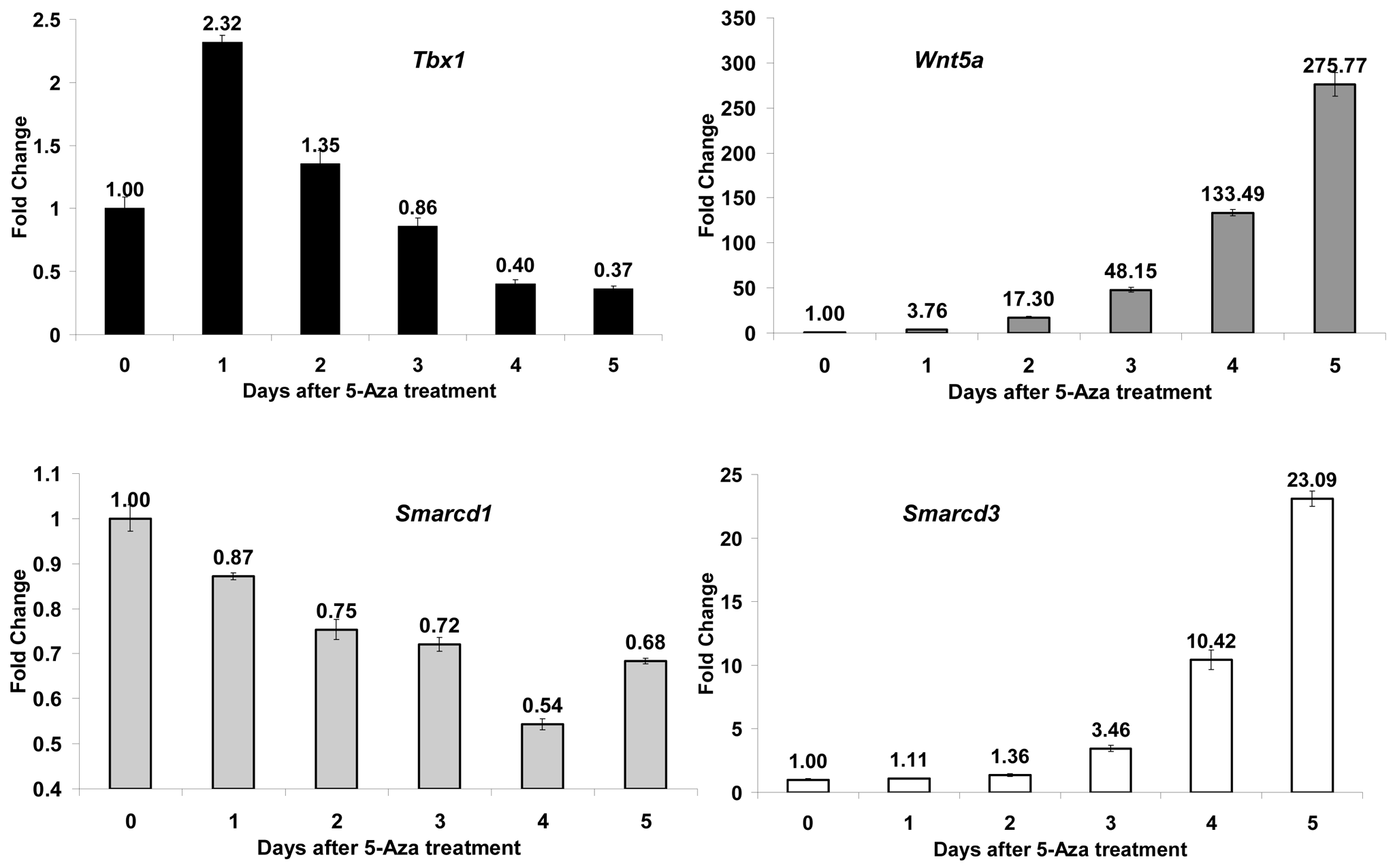

Supplement: Figure S1 — Expression analyses by quantitative real time PCR of genes Tbx1, Wnt5a, Baf60a/Smarcd1 and Baf60c/Smarcd3 in undifferentiated P19Cl6 cells (day 0) and during the first 5 days of treatment to induce differentiation. (TIF) [file pgen.1002571.s001.tif]

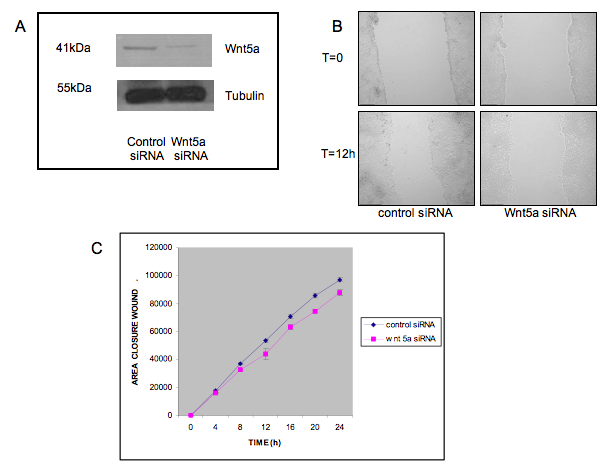

Supplement: Figure S2 — In vitro, wound healing assay performed on P19Cl6 cells before and after Wnt5a knock-down by siRNA. (A) Western blot demonstrating knock-down of Wnt5a expression. (B) Microphotographs of the wound healing assay on P19Cl6 cells. (C) Summary of results expressed as area of closure with and without Wnt5a over time, up to 24 hours. Data were obtained by time-lapse microscopy. Control cells recover a significantly larger area after the wound (P = 0.01, t-test). (TIF) [file pgen.1002571.s002.tif]

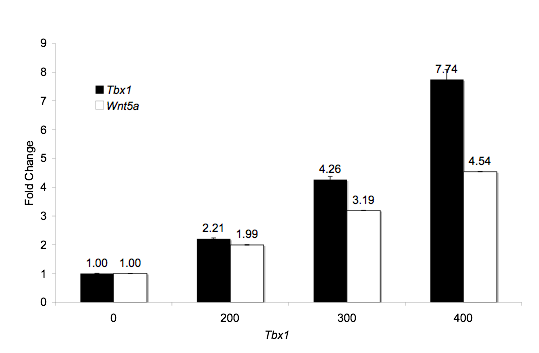

Supplement: Figure S3 — Tbx1 and Wnt5a gene expression analysis by quantitative real time PCR assay in cells expressing Tbx1 after transfection with the indicated amounts of a Tbx1 expression vector. Each data point is expressed as the mean of three replicates ± SD. (TIF) [file pgen.1002571.s003.tif]

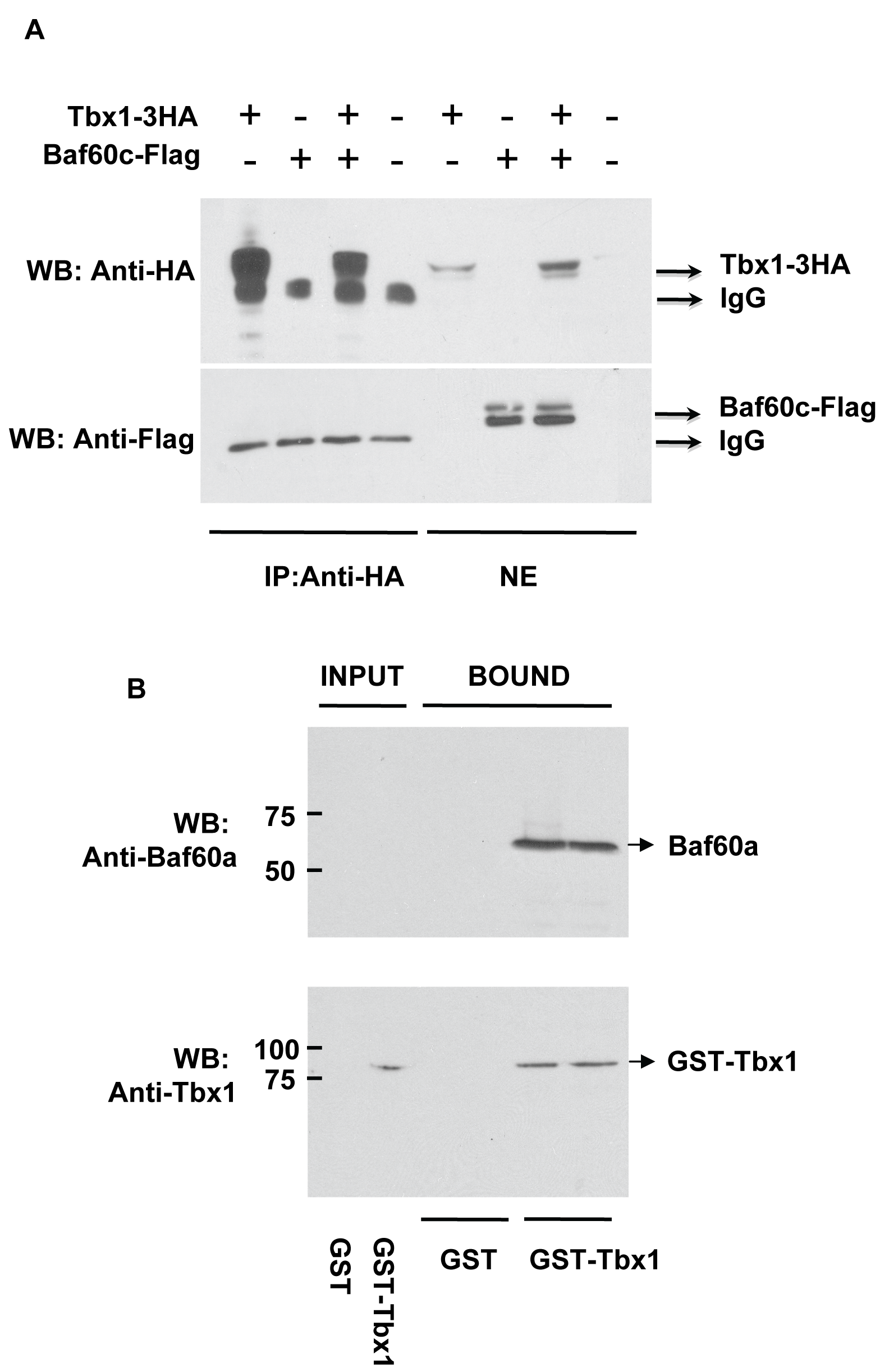

Supplement: Figure S4 — (A) Western blot analyses of coimmunoprecipitation experiments using the anti-HA-immunoprecipitation kit. P19Cl6 cells were co-transfected transiently with Tbx1-3HA and Baf60c-Flag expression vectors). Western blots were carried out using anti-HA and anti-Flag antibodies. NE: nuclear extracts (10% input). (B) Direct interaction assay using GST pull-down shows that Tbx1 interacts with Baf60a. Western blot analysis of GST-Tbx1 pull-down from a lysate of P19Cl6 cells overexpressing Baf60a. The input lane shows Tbx1 expression in the lysate. (TIF) [file pgen.1002571.s004.tif]

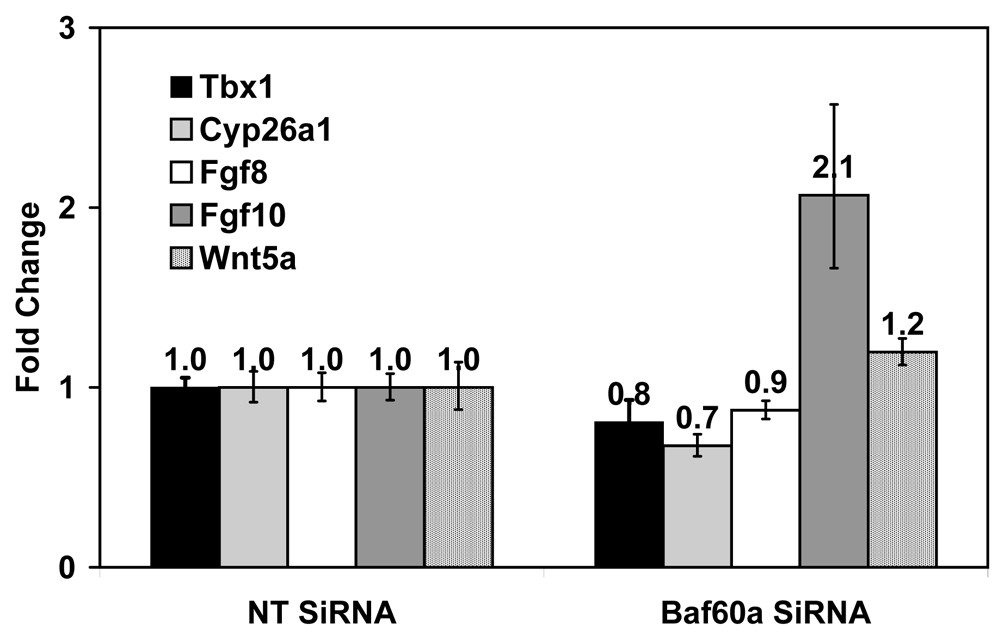

Supplement: Figure S5 — Quantitative real-time PCR evaluation of the expression of Tbx1, Cyp26a1, Fgf8, Fgf10, and Wnt5a with and without Baf60a knock-down by siRNA in undifferentiated P19Cl6 cells. Note that the knock-down of Baf60a has no significant effect on the expression of these genes (P-value more than 0.052. (TIF) [file pgen.1002571.s005.tif]
